# Supplementary material for: Content-rich biological network constructed by mining PubMed abstracts
Source: BMC Bioinformatics. 2004 Oct 8;5:147. doi: 10.1186/1471-2105-5-147 (PMC528731; doi:10.1186/1471-2105-5-147)
Supplement: Additional File 2 — The original results of the above study (non-essential files are deleted to keep the file size under the limit set by BMC bioinformatics). [file 1471-2105-5-147-S2.bz2 › chilibotAdditionalFile2/dip20/489ID10400640E10649/html/AR_RAN.html]

 


 **AR** and **RAN** 
  
Found 12 abstracts in PubMed, retrieved 12.  
 

 What does Google say? 
 PDF only 
| .edu only 

---

**Interactive relationship** (e.g. stimulation, inhibition, etc)

**Neutral relationship**- Here we have identified  **Ran**  binding protein in the microtubule organizing center RanBPM to be a novel  **AR**  interacting protein by yeast two hybrid assay and have confirmed this interaction by glutathione S transferase and His tagged pull down assays.  Ref: 12361945 J Biol Chem, 2002
- **AR**   **ran**  more miles day and consumed fewer kcal d to support energy expenditure compared to ER p <.  Ref: 8325717 Int J Sports Med, 1993
- Our data indicate that ARA24  [ **RAN** ]  could also interact with  **AR** ,.  Ref: 11155740 J Formos Med Assoc, 2000
- In the yeast and mammalian reciprocal interacting assays, our data suggested the interaction of  **AR**  N terminal domain with ARA24  [ **RAN** ]  diminishes as the poly Q length increases.  Ref: 10400640 J Biol Chem, 1999
- The coactivation of ARA24  [ **RAN** ]  also diminishes with the poly Q expansion within  **AR** .  Ref: 10400640 J Biol Chem, 1999
- In addition to the  **AR**  LBD associated proteins, ARA24  [ **RAN** ]  and ARA160 have been identified as  **AR**  coregulators, interacting with the  **AR**  N terminal instead of the LBD.  Ref: 11155740 J Formos Med Assoc, 2000
- Together, our data suggest that poor interaction and weaker coactivation of ARA24  [ **RAN** ]  to the longer poly Q  **AR**  in the X linked spinal and bulbar muscular atrophied  **AR**  could contribute to the weaker transactivation of  **AR** .  Ref: 10400640 J Biol Chem, 1999
- Here we report the identification of a nuclear G protein, Ras related nuclear protein  [ **RAN** ]  ARA24  [ **RAN** ] , as the first  **AR**  coactivator that can bind differentially with different lengths of poly Q within  **AR** .  Ref: 10400640 J Biol Chem, 1999
- Several new androgen receptor  [ **AR** ]   **AR**  coregulators, including ARA70, ARA55, ARA54, ARA160 and ARA24  [ **RAN** ] , associated with the N terminal or the ligand binding domain LBD of  **AR** , have been identified by our group.  Ref: 11155740 J Formos Med Assoc, 2000

**Non-interactive relationship** (e.g. studied together, co-existance, homology, etc.)

- In  **AR** , the rats  **ran**  at a speed of 25 m.min 1 for 20 min after injection of U 14C lactate.  Ref: 3253233 Int J Sports Med, 1988
- Another  **AR**  N terminal interacting protein, ARA24  [ **RAN** ] , interacted with the poly Q tract, a region within the N terminus of the  **AR**  linked to Kennedy s disease X linked spinal and bulbar muscular atrophy.  Ref: 11501969 J Biol Regul Homeost Agents< MedlineTA>J Biol Regul Homeost Agents, 1988
- Our results revealed nearly constant expression of  **AR**  and heterogeneous expression of  **AR**  co factors, with increased expression of PIAS1 and  **Ran**  ARA24  [ **RAN** ] , decreased expression of ELE1 ARA70, and no change in TMF1 ARA160, ARA54, SRC1, or TRAP220.  Ref: 12368219 Am J Pathol, 2002
- ARA267 alpha can also enhance  **AR**  transactivation with other coregulators, such as ARA24  [ **RAN** ]  or PCAF, a histone acetylase, in an additive manner.  Ref: 11509567 J Biol Chem, 2001
- Deletion of the acidic hexapeptide DEDDDL at the C terminus of ARA24  [ **RAN** ]  further enhances its  **AR**  coactivation.  Ref: 10400640 J Biol Chem, 1999
- The linkage of Kennedy s neuron disease to ARA24  [ **RAN** ] , the first identified androgen receptor  [ **AR** ]  polyglutamine region associated coactivator.  Ref: 10400640 J Biol Chem, 1999
- We demonstrate by DNA microarray, RT PCR, Western blot and immunofluorescence analyses that treatment of androgen sensitive prostate cancer cells LNCaP with 10 5 M RE for 48 hr downregulates prostate specific antigen PSA,  **AR**  co activator ARA 24  [ **RAN** ]  and NF kB p65.  Ref: 12569576 Int J Cancer, 2003
